# Responses of Ephemeral Plants to Precipitation Changes and Their Effects on Community in Central Asia Cold Desert

**DOI:** 10.3390/plants12152841

**Published:** 2023-08-01

**Authors:** Xiaohan Mu, Xinjun Zheng, Gang Huang, Lisong Tang, Yan Li

**Affiliations:** 1 State Key Laboratory of Desert and Oasis Ecology/Fukang Station of Desert Ecology, Xinjiang Institute of Ecology and Geography, Chinese Academy of Sciences, Urumqi 830011, China; muxiaohan@ms.xjb.ac.cn (X.M.);; 2 School of Geographical Sciences, Fujian Normal University, Fuzhou 350007, China

**Keywords:** trait correlation networks, desert, drought, precipitation change, survival strategy

## Abstract

In the context of global climate change, changes in precipitation patterns will have profound effects on desert plants, particularly on shallow-rooted plants, such as ephemeral plants. Therefore, we conducted an experiment on artificial control of precipitation for four dominant ephemeral plants, *Erodium oxyrhinchum*, *Alyssum linifolium*, *Malcolmia scorpioides*, and *Hyalea pulchella*, in the southern edge of Gurbantunggut Desert. We measured the importance value and some growth parameters of the four species under increased or decreased precipitation and constructed trait correlation networks for each of the four species. We also compared the response of increased or decreased precipitation to vegetation coverage. The results show that drought significantly reduced the survival rate, seed production and weight, and aboveground biomass accumulation of ephemeral plants. The four ephemeral plants showed different tolerance and response strategies to precipitation changes. *E. oxyrhinchum* and *M. scorpioides* can avoid drought by accelerating life history, and *E. oxyrhinchum*, *A. linifolium,* and *H. pulchella* can alleviate the negative effects of drought by adjusting leaf traits. However, the response of different species to the wet treatment was not consistent. Based on the results of the trait correlation network, we consider *A. linifolium* belongs to the ruderal plant, *E. oxyrhinchum* and *M. scorpioides* belong to the competitive plants, and *H. pulchella* belongs to the stress-tolerant plant. The outstanding trait coordination ability of *E. oxyrhinchum* makes it show absolute dominance in the community. This indicate that ephemeral plants can adapt to precipitation changes to a certain extent, and that distinct competitive advantages in growth or reproduction enabled species coexistence in the same ecological niche. Nevertheless, drought significantly reduces their community cover and the ecological value of ephemeral plants. These findings established the basis to predict vegetation dynamics in arid areas under precipitation changes.

## 1. Introduction

According to global climate scenarios predictions, precipitation patterns in arid areas will change significantly [1]. Extreme events such as droughts or heavy rains have become more frequent, with greater interannual and seasonal variability, which has altered the water balance of terrestrial ecosystems [2,3]. The desert ecosystem is one of the most sensitive ecosystems to precipitation; thus, the changes in water availability and frequent extreme drought events caused by climate change have a profound impact on the survival of desert vegetation [4,5].

Ephemeral plants are the main contributors to species diversity and herbaceous layer productivity, playing an important role in the succession in northern temperate desert plant communities [6]. More than 40 ephemeral plants species in Gurbantungut Desert, central Asia, accounting for approximately one third of the desert’s total plant species [7,8]. This biologically diversified and wide distribution of plant types helps to improve soil nutrient cycles, decrease the extent of desertification and sandstorms, and thus increase ecosystem stability in Gurbantungut Desert [9,10,11]. Ephemeral plants also support the development and security of the surrounding agricultural and urban ecosystems; however, current environmental problems pose an increasing threat to these plant communities [12].

In arid regions, precipitation is the main driving force controlling above-ground net primary production, community structure and plant composition [13,14]. The growth and development of desert plants are primarily limited by precipitation, which drives many key ecological and physiological processes, notably seed germination, ontogenesis, and biogeochemical cycles [15,16,17,18]. The desert’s low total precipitation [19], coupled with warming and precipitation changes caused by global climate change have led to fluctuations in shallow soil water content, while ephemeral plants with shallow-rooted lack a stable water source and are therefore vulnerable to drought stress [20,21].

Desert plants exhibit considerable adaptability and self-regulation strategies to changes in water conditions [22,23,24]. Some species have specialized leaves and well-developed root systems, others actively adjust organ biomass allocation [25,26]. However, even species in the same ecological niche may have different coping strategies in the face of changes in their resource environment, and we do not know enough about this, especially for abundant ephemeral plants [27,28]. In addition, the changes in resource availability aggravate competition for resources and negatively affect interactions between species, which may lead to changes in overall community patterns [29,30,31]. Previous studies on the consequences of changes in water availability in arid areas have generally focused on biodiversity and biomass production [32,33], while studies on population survival strategies were limited to individual dominant species [34]. Therefore, the aim of this research is to understand different plant survival strategies and elucidate the effects of precipitation on community development, particularly in under-researched regions such as central Asia. This study will contribute to forecasting the future dynamics of temperate ecosystems under global change.

To better understand the effects of fluctuations in precipitation on growth of ephemeral plant populations and the survival strategies of different plants, we experimentally controlled the growing season precipitation (50% reduced, 100% increased) in the Gurbantunggut Desert in 2017. We hypothesized that precipitation change would affect ephemeral plants survival and growth parameters, while they would exhibit different survival strategies, which would affect their respective position in the community and the ecological function of the community.

## 2. Results

### 2.1. Effects of Precipitation Changes on Plant Survival and Growth

**Figure 1:**
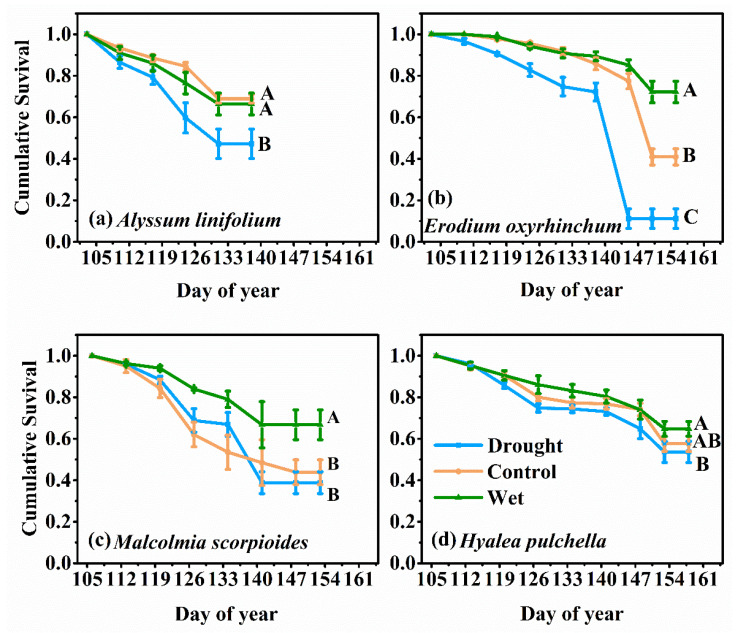
Kaplan–Meier survival analysis of *Alyssum linifolium* (**a**), *Erodium oxyrhinchum* (**b**), *Malcolmia scorpioides* (**c**), and *Hyalea pulchella* (**d**) under the drought (50% reduction in precipitation), control, or wet (100% increase in precipitation) treatment. Different capital letters indicate significant differences between treatments (*p* < 0.05).

Kaplan–Meier survival analysis showed that the survival rates of the four ephemeral plants were significantly reduced under drought treatment (*p* < 0.05, Figure 1). The survival rate of *A. linifolium* was reduced to 47.2% under drought treatment, which was significantly lower than that of the other treatments. In the wet treatment, the final survival rate of *E. oxyrhinchum* reached up to 72.2%, which was the highest among the four species. However, *E. oxyrhinchum* was also the most strongly affected by the drought treatment among the four species, and the final survival rate was as low as 11.2%. Under the wet treatment, the survival rates of *M. scorpioides* and *H. pulchella* were significantly increased to 66.74% and 64.78%, respectively. Plant survival was not largely affected by precipitation changes in the early growth stage. Plants showed sensitivity to precipitation changes in the middle and late growth stages, especially to the drought treatment (Figure 1).

The lifetimes of the four species were significantly different (F = 203.409, *p* < 0.05). *A. linifolium* had the shortest lifetime of about 39 days (Table 1). The rest of the species are longer lived, but drought significantly shortened the lifetime of *E. oxyrhinchum and M. scorpioides* (*p* < 0.05) (Table S1). *H. pulchella* can survive about 56 days, and its lifetime was not affected by precipitation changes.

**Figure 2:**
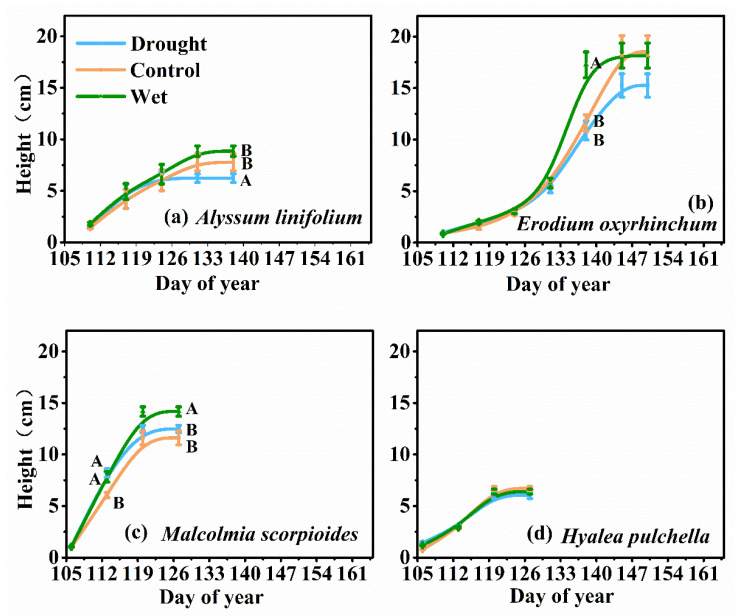
Trends in growth (measured according to height) of *Alyssum linifolium* (**a**), *Erodium oxyrhinchum* (**b**), *Malcolmia scorpioides* (**c**), and *Hyalea pulchella* (**d**) plants under drought (50% reduction in precipitation), control, or wet (100% increase in precipitation) treatment. Different capital letters indicate significant differences between treatments (*p* < 0.05).

The height of the four species was significantly different (F = 136.93, *p* < 0.05, Figure 2). Precipitation treatments had a significant effect on the final average height of the *A. linifolium* populations and *M. scorpioides* (*p* < 0.05), but had no effect on *E. oxyrhinchum* and *H. pulchella*. At the late stage of plant growth, the wet treatment significantly increased the growth rate of *E. oxyrhinchum* and *M. scorpioides*.

### 2.2. Effect of Precipitation Change on Plant Production

**Figure 3:**
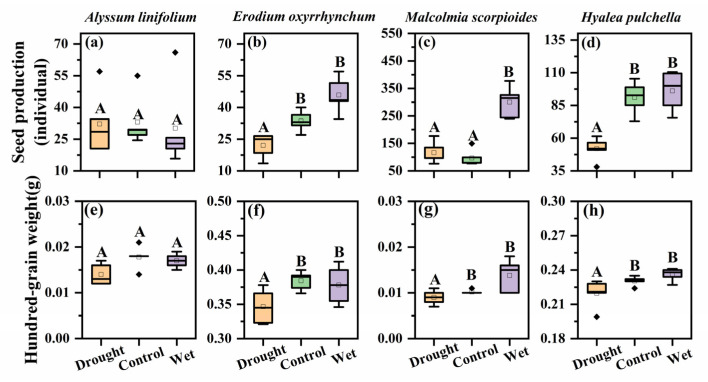
Seed production and of hundred-grain weight *Alyssum linifolium* (**a**,**e**), *Erodium oxyrhinchum* (**b**,**f**), *Malcolmia scorpioides* (**c**,**g**), and *Hyalea pulchella* (**d**,**h**) plants under drought (50% reduction in precipitation), control, or wet (100% increase in precipitation) treatment. Different capital letters indicate significant differences between treatments (*p* < 0.05). Boxes indicate the upper (75%) and lower (25%) quartiles. Whiskers represent the ranges of the minimum and maximum values. The points outside whiskers are statistical outlier values.

The seed production significantly differed among the four species (F = 29.49, *p* < 0.05, Figure 3). *M. scorpioides* had the highest seed production, and its seed production was greatly improved under wet treatment, up to about 300 seeds per plant (*p* < 0.05). At the same time, the seed production of *E. oxyrhinchum* and *H. pulchella* was significantly reduced under drought treatment (*p* < 0.05), were about 22 seeds per plant and 116 seeds per plant, respectively. However, the seed production of *A. linifolium* was not affected by precipitation treatment.

The hundred-grain weight significantly differed among the four species (F = 2527.13, *p* < 0.05, Figure 3). Among them, the seeds of *A. linifolium* and *M. scorpioides* were extremely light, about 0.01–0.02 g per hundred grain, whereas the seeds of *E. oxyrhinchum* were heavier, about 0.30–0.40 g per hundred grain. The hundred-grain weight of *E. oxyrhinchum*, *M. scorpioides,* and *H. pulchella* were significantly reduced under drought treatment (*p* < 0.05).

### 2.3. Effect of Precipitation Change on Plant Aboveground Biomass and Leaf Traits

**Figure 4:**
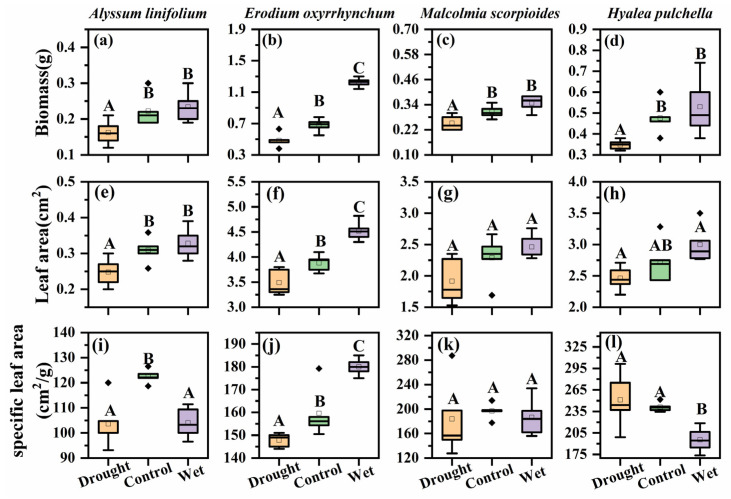
Aboveground biomass, leaf area and specific leaf area of *Alyssum linifolium* (**a**,**e**,**i**), *Erodium oxyrhinchum* (**b**,**f**,**j**), *Malcolmia scorpioides* (**c**,**g**,**k**), and *Hyalea pulchella* (**d**,**h**,**l**) plants under drought (50% reduction in precipitation), control, or wet (100% increase in precipitation) treatment. Different capital letters indicate significant differences between treatments (*p* < 0.05). Boxes indicate the upper (75%) and lower (25%) quartiles. Whiskers represent the ranges of the minimum and maximum values. The points outside whiskers are statistical outlier values.

The aboveground biomass also significantly differed among the four species (F = 50.68, *p* < 0.05, Figure 4). *A. linifolium* and *H. pulchella* had lower aboveground biomass compared with that of *E. oxyrhinchum*. The precipitation treatments had a significant effect on the aboveground biomass of all species (*p* < 0.05), which significantly decreased in the drought treatment. The above-ground biomass of *E. oxyrhinchum* increased under the wet treatment, whereas this treatment had no significant increased on the biomass of the other species.

The leaf area (F = 466.81, *p* < 0.05, Figure 4) and specific leaf area (F = 50.11, *p* < 0.05, Figure 4) of the four species were significantly different. The leaf area of *E. oxyrhinchum* was the largest. Precipitation treatment significantly affected the leaf area of *E. oxyrhinchum*, *A. linifolium*, and *H. pulchella*, which mainly manifested as a decrease in leaf area under drought treatment, whereas the leaf area of *M. scorpioides* was not affected by precipitation treatment (*p* < 0.05). Drought treatment significantly reduced the specific leaf area of *E. oxyrhinchum*, *A. linifolium*, and *H. pulchella*, but not that of *M. scorpioides* (*p* < 0.05).

### 2.4. Trait Correlation Networks for Four Plant Species

**Figure 5:**
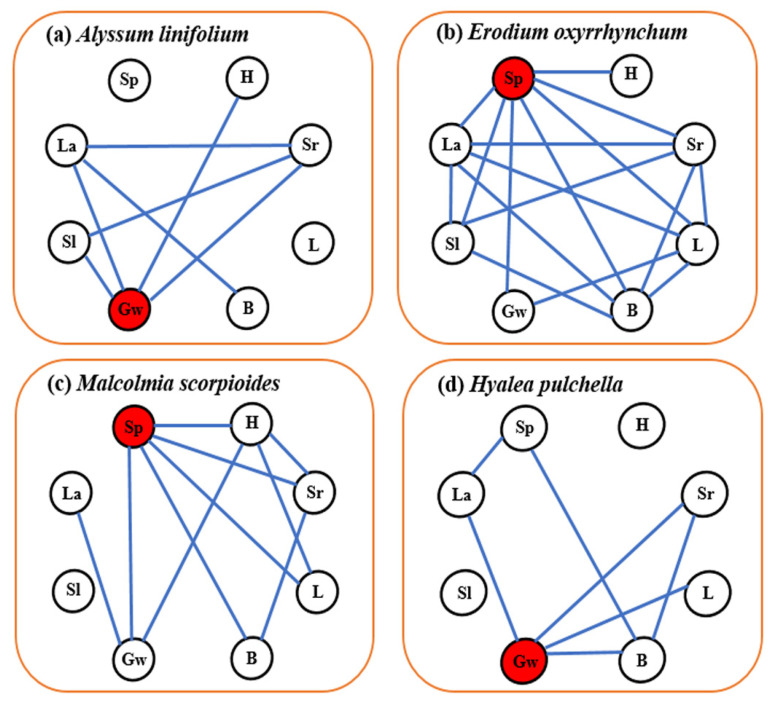
Trait correlation networks of *Alyssum linifolium* (**a**), *Erodium oxyrhinchum* (**b**), *Malcolmia scorpioides* (**c**), and *Hyalea pulchella* (**d**). Only significant correlations are shown (*p* < 0.05). Traits identified by red circle show the central trait. The abbreviations mean: Sp: seed production; H: height; Sr: survival rate; L: lifetime; B: aboveground biomass; Gw: hundred-grain weight; Sl: specific leaf area; La: leaf area.

*E. oxyrhinchum* had the most complex trait correlation network, seed production had the closest relationship with other traits in the networks with the number of edges of the trait network was 7 (Table S2), and was the central trait (Figure 5). Seed production were the central traits with the number of edges of the trait network was 5 of *M. scorpioides*. The trait correlation network of *A. linifolium* and *H. pulchella* were relatively simple, hundred-grain weight was their central trait, and the total number of edges of the trait network was seven for both species.

### 2.5. Effect of Precipitation Change on Importance Values and Vegetation Coverage

**Figure 6:**
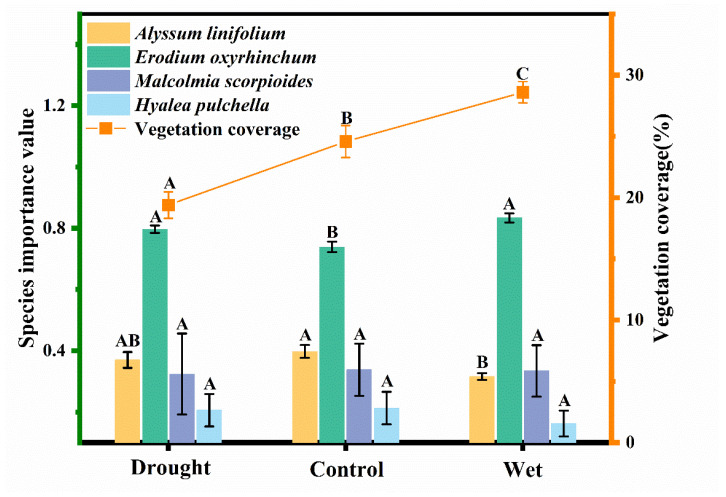
Species importance value of *Alyssum linifolium*, *Erodium oxyrhinchum*, *Hyalea pulchella*, and *Malcolmia scorpioides* (left *Y*-axis) and vegetation coverage of the ephemeral plants layer (right *Y*-axis) under drought (50% reduction in precipitation), control, or wet (100% increase in precipitation) treatment. Different capital letters indicate significant differences between treatments (*p* < 0.05).

Significant differences in importance values were found among species, with the highest value observed for *E. oxyrhinchum*, followed by *A. linifolium*, *M. scorpioides*, and *H. pulchella* (F = 62.42, *p* < 0.05, Figure 6). The importance value of *A. linifolium* decreases with wet treatment, while the importance value of *E. oxyrhinchum* decreases with control (*p* < 0.05). However, the importance values of other species were not affected by the precipitation treatment. Precipitation treatment significantly affected the coverage of the ephemeral plants layer, and the increase in water resulted in a significant increase in coverage (F = 17.59, *p* < 0.05, Figure 6).

## 3. Discussion

### 3.1. Effect of Precipitation Changes on Ephemeral Plants

Water is indispensable for plant growth and development, mitigate drought and contribute the establishment of seedlings [35,36]. The survival of ephemeral plants in the early life cycle was not affected by precipitation treatment (Figure 1), this is because the early spring snow melt in Gurbantunggut Desert greatly replenishes soil moisture, which provides the necessary water for seedling growth [37]. The survival of ephemeral plants began to decrease to varying degrees over time, and the wet treatment had a significant effect on alleviating the mortality of the plants (Figure 1). This implies that as snowmelt water was depleted and temperatures gradually increase, ephemeral plants become more dependent on precipitation, while increased precipitation improves the arid desert environment and increases plant survival rate [38]. The large biomass and leaf area of the *E. oxyrhinchum* may explain its higher water sensitivity in the later stages of growth (Figure 4). It can be concluded that ephemeral plants have a strong dependence on precipitation, and the trend of increasing precipitation in the future will favor the growth of desert ephemeral plants [39].

Water can limit the accumulation of biomass in plants [40], but is not the only factor. In this study, the plant height of *A. linifolium* (Figure 2) decreased significantly under the drought treatment, and all plants showed varying degrees of reduction in aboveground biomass accumulation (Figure 4). Similar results were found in related studies, in which ephemeral plants showed significantly lower height and biomass in a dry spring than in a wet spring [41]. Drought stress may cause stomatal closure and decreased enzyme activity, which may limit the photosynthetic capacity of plants and reduce the accumulation of dry matter. However, there was no general increase in plant height and aboveground biomass with increased precipitation (Figure 2 and Figure 4). We believe that this was related to competition for resources, in addition to genetic factors of the species. Adequate water increases plant survival and density (Figure 1), but the resources available in an ecosystem are limited, especially in deserts. High population densities increase competition between plants for water and nutrients, and even space [42,43]. Fan [7] also confirmed a negative correlation between plant size and plant density in a study of the limiting effect of snowpack on desert plants. Therefore, we think that in desert ecosystems, water is the first limiting factor for population growth, and after that, the effects of nutrients and interspecific relationships should also be considered.

Seed production and hundred-grain weight are important traits and standard indicators of plant reproductive capacity, which are also closely related to the nutritional status of plants [44,45]. In our study, precipitation would directly affect seed production and the hundred-grain weight of *E. oxyrhinchum*, *M. scorpioides* and *H. pulchella* (Figure 3). Previous studies have repeatedly demonstrated that drought stress causes low carbohydrate production, low fruit set and pollen sterility in plants, leading to significant decreases in yields of crops such as corn, soybean and sunflower [46,47,48]. Reproduction is the most important stage in the life of a plant, and seeds are the main carriers of ephemeral plant population dispersal and persistence through the harsh desert environment [49,50]. It follows then that prolonged drought stress will affect the recovery of ephemeral plants populations and the survival of seedlings in the coming year [51,52].

### 3.2. Adaptation Strategies of Ephemeral Plants to Changes in Precipitation

Ephemeral plants are drought-avoiding plants whose survival strategy is to grow rapidly and complete their life history before the onset of the hot summer [53]. In this study, we found that *E. oxyrhinchum* and *M. scorpioides* accelerated life cycle completion during drought and increased growth rate during wet treatment (Table 1 and Figure 2). These results are consistent with previous studies on the community composition and phenology of ephemeral plants, indicating that precipitation has a decisive effect on the life history of ephemeral plants, and sufficient water can promote the growth of ephemeral plants and induce changes in plant phenology [11,54]. Overall, ephemeral plants can adapt to changing environmental conditions by regulating their life cycles and growth rates, a growth pattern that gives them a greater advantage in surviving in the changing environment of the future.

Indeed, ephemeral plants have highly plasticity to cope with the changeable and unpredictable desert environment [55,56]. The leaf morphology of plants is closely related to their physiological needs, and a change in leaf morphology is a manifestation of adaptation to different habitats [57,58]. In this study, under drought treatment, the leaf area and specific leaf area of *E. oxyrhinchum*, *A. linifolium*, and *H. pulchella* decreased to varying degrees (Figure 4), which can help to reduce carbon depletion for building and upkeep the stems and leaves, meanwhile to reduce transpiration and improve the water storage capacity [59,60]. Adjustment of these plant functional traits is beneficial to adapt to the desert environment characterized by drought, torridity and strong radiation, and indicate the trade-off between adaptation to the environment and optimal function of ephemeral plants, thereby maximizing resource use and energy accumulation while ensuring survival [56].

The different responses of these species to precipitation changes and different trait correlation networks support our hypothesis that ephemeral plants have different survival strategies. *A. linifolium* has the smallest biomass, small leaf area and specific leaf area, and has the shortest life span and was not associated with other traits. These characteristics not only reduce the costs of growth, ensure seed quality, but also facilitate rapid completion of the life cycle, thereby greatly reducing the risks of drought [59]. This is consistent with what we have previously considered to be a drought avoidance strategy for ephemeral plants [11] and we consider this survival strategy of rapid access to resources for growth and rapid reproductive success to be a ruderal plant [61]. *E. oxyrhinchum* positively regulates leaf area, specific leaf area and life history to ensure smooth reproduction in drought treatment; and obviously increases reproductive investment when water was sufficient [39,54]. At the same time, the complex trait network of *E. oxyrhinchum* shows that it has strong coordination and high adaptability to desert. *M. scorpioides* showed high reproductive capacity under adequate water conditions by increasing inputs to the aboveground parts, such as height and biomass. This is consistent with the results that ephemeral plants tend to allocate more biomass to their reproductive organs and improve reproductive success through high seed production [62]. This strategy of *E. oxyrhinchum* and *M. scorpioides* to maximize the use of resources to increase population fecundity can be considered as competitive plants. The relatively simple trait coordination network of *H. pulchella* indicates that it has low adaptability to desert environment, but it still maintains a high hundred-grain weight through the coordination of multiple traits, so it was stress-tolerant plant.

The above conclusions indicate that ephemeral plants have a certain ability to adapt and self-regulate to the desert environment. At the same time, these trade-offs between high growth rates and competitive tolerance of these four ephemeral plant species that are dominant in Gurbantunggut Desert help to stabilize species coexistence, thus explaining the coexistence of species in the same niche [63,64]. Thus, these distinct characteristics of the four species expand the overall guild characteristics and niche breadth, which is beneficial to maintaining community complexity and stability [65,66].

### 3.3. Effects of Precipitation Changes on the Herbaceous Layer Pattern

Our results showed that *E. oxyrhinchum* had the highest importance value and this position was not affected by precipitation (Figure 6), reflecting its absolute superiority over other species, which was consistent with previous studies [25,34]. In addition, this must be inseparable from its excellent trait coordination ability with seed production as the central trait (Figure 5). The wet treatment did not significantly enhance the population survival (Figure 1) and individual growth (Figure 4) of *A. linifolium*, but promoted the growth of other plants to a certain extent, resulting in an overall decrease in the importance value of *A. linifolium*. Notably, we found that the ephemeral plants differed in their sensitivity to water (Figure 1), and when the resource match changes too drastically, competition between species intensifies [30]. Species that are more sensitive to environmental changes or those that exhibit slow growth may face unprecedented pressure from fast-growing and more competitive species. Therefore, the absolute advantage of *E. oxyrhinchum* means that establishing populations of other species is quite difficult, which would negatively impact the stability of biodiversity and community structure. 

Wind is the main force of desert erosion, and ephemeral plants play an important role in slowing down the rate of erosion [67,68,69]. At the same time, ephemeral plants play an important role in nutrient retention and recycling in desert soils [70,71]. Our study not only confirms the critical role of water on vegetation cover (Figure 6), but also demonstrates the strategies of ephemeral plants to shorten their life cycle and adjust functional traits to cope with drought stress (Figure 1 and Figure 4 and Table 1). These results confirm our hypothesis that the adaptation strategies of ephemeral plants to drought will directly affect their ecological functions of windbreak and sand fixation and nutrient cycling, thus affect the stability of desert ecosystem [72,73].

## 4. Methods

### 4.1. Study Site

The study site is located in the vicinity of the Fukang Station of Desert Ecology (44°17′N, 87°56′E, 475 m a.s.l.), Chinese Academy of Sciences, at the southern edge of Gurbantunggut Desert in the hinterland of the central Asia. This area has a typical temperate continental climate with four distinct seasons. The average annual temperature here is 6.6 °C (1997–2016), the coldest monthly average temperature −15 °C (January), the hottest 20 °C (July) [7]. The desert is extremely arid with annual precipitation of 160 mm and a 30-cm-deep stable seasonal snowpack in winter [74]. Spring snowmelt is an important water source for the germination of desert plants. Haloxylon ammodendron and Haloxylon persicum are the group species of this ecosystem, along with a number of herbaceous plants, such as Ceratocarpus arenarius and Nepeta micrantha. There are 40 species of ephemeral plants present in the study area. Most of these plants are members of the families Brassicaceae and Asteraceae, and the common species recorded are Erodium oxyrhinchum and Alyssum linifolium.

### 4.2. Experimental Design

*Erodium oxyrhinchum* (Geraniaceae), *Alyssum linifolium* (Brassicaceae), *Malcolmia scorpioides* (Brassicaceae), and *Hyalea pulchella* (Compositae) were selected as the plants of focus for this study. These species are known to germinate in late March or early April, after the snow has melts, and they all adopt a rapid growth strategy to escape the hot and dry summer, resulting in a short lifespan of only 40–60 d. In addition, they are widely distributed in the region and are very easy to observe and harvest for study.

April–June is the growing season for ephemeral plants in the Gurbantunggut desert, with an average precipitation of 61.7 mm (2005–2016). In fact, the interannual fluctuations of growing season precipitation were large, with a maximum value of 86.9 mm in 2016 and a minimum value of 22.9 mm in 2012 (all precipitation data in this study were obtained by an automatic weather station (Campbell Scientific, Logan, UT, USA) installed near the study site by Fukang Station of Desert Ecology). According to precipitation statistics from weather stations, the total rainfall from March to June 2017 was 78.4 mm. In this study, we set up three treatments: drought (50% reduction in precipitation, 39.2 mm), wet (100% increase in precipitation, 156.8 mm), and control (natural precipitation). There were five replicates per treatment, consisting of adjacent 10 m × 10 m quadrats, which can be found in the graphical abstract. The rainout shelter of drought treatment was composed of 10 m × 10 m steel frame and transparent polycarbonate plastic strips. Transparent polycarbonate plastic strips are evenly spaced fixed the steel frame, covering 50 percent of the area and just enough to reduce rain by 50 percent. The trapped rainwater flows through polyvinyl chloride pipes into a collection bucket. We collected additional precipitation, which was used along with the rainwater collected by the drought treatment, to increase precipitation for the wet treatment. Please refer to Figure S1 for schematics of experimental design and photos of species.

The experiment began in mid-April, a week after the snow melted and the plants began to sprout. We labeled and numbered 300–600 individuals of *E. oxyrhinchum*, *A. linifolium*, *M. scorpioides*, and *H. pulchella* for each treatment, depending on their population densities. The inner edge of the 1-m sample square was used as a buffer zone and a destructive sampling zone was delineated.

### 4.3. Measurement and Sampling

#### 4.3.1. Survivorship, Lifetime and Plant Height

We observed and measured the labeled plants once a week starting in mid-April, recording their height (measured with a ruler) and survival status until the plants died or completed their life cycle. At the same time, in each sample, five of each species were selected for observation and used to count the lifetime of the species.

#### 4.3.2. Seed Production and Hundred-Grain Weight

Most ephemeral plants begin to flower and produce seeds in May. However, seeds fall off easily when they mature, making it difficult to quantify the number of seeds. Therefore, we directly counted the number of capsules per plant before seed maturity (in mid-May), and then multiplied by the number of seeds in the capsule to obtain the total seed production per plant. The seeds were collected after maturity and brought back to the laboratory to dry naturally, where they were weighed in groups of 100 seeds using an electronic balance (Ohaus, NJ, USA), calculated as the hundred-grain weight of the species.

#### 4.3.3. Leaf Area, Specific Leaf Area and Aboveground Biomass

At the peak of plant growth (the peak times varies from species to species), harvest two plants per species in the destructive sampling area. We first remove the leaves from the plant and then use the scanner (Epson Perfection 2400 Photo, Seiko Epson, Nagano, Japan) to calculate the leaf area (Computer Imaging Analysis Software, CID Co., Logan, UT, USA). The leaves, along with the whole plant, were then placed in an oven and dried at 75 °C for 48 h, and the aboveground biomass of the plant was weighed using a balance (Ohaus). Finally, we use a balance to weigh the leaves separately and get the specific leaf area by leaf area/leaf weight.

#### 4.3.4. Importance Value and Vegetation Coverage

In the period of vigorous plant growth in early May, a 1 m × 1 m quadrat was marked out from each quadrat to determine the quadrat vegetation coverage and importance value of the studied species. The importance value was calculated as (relative density + relative coverage + relative height)/3, where relative density = number of individuals of the species/total number of all plants, relative cover (square grid method) = cover of the species/total cover of all plants, and relative height = (average height of the species/average height of the dominant species) × 100%. The dominant species was *E. oxyrhinchum*.

#### 4.3.5. Trait Coordination Networks

Trait coordination networks of different species were described by statistically significant correlations among traits (including leaf area, specific leaf area, aboveground biomass, seed production, hundred-grain weight, survival rate, plant height, and lifetime) and illustrated using the Igraph package in R (R 3.5.3). The number of its adjacent edges determine the network centrality.

### 4.4. Statistical Analyses

A linear mixed model was performed to analyze the differences in plant height, aboveground biomass, seed production, hundred-grain weight, leaf area, specific leaf area, and importance value among species and precipitation treatments. One-way analyses of variance (ANOVAs) were performed to analyze the differences in plant height, lifetimes, aboveground biomass, seed production, hundred-grain weight, leaf area, specific leaf area, and importance value of single species under different precipitation treatments, as well as the differences in vegetation coverage under different precipitation treatments. The Kaplan–Meier method was used to analyze the survival analyses of each species. *p* < 0.05 was used to assess the differences between treatments. Origin 9.0 (OriginLab Corp., Northampton, MA, USA) was used to generate figures.

## 5. Conclusions

This study shows that the phenomenon of increased precipitation variability due to global climate change can directly affect the survival, growth, reproduction, and functional traits of ephemeral plants in a desert environment. However, ephemeral plants can alleviate the adverse effects of environmental change by accelerating growth and regulating leaf traits. At the same time, ephemeral plants exhibit species-specific differences in coping strategies and growth characteristics, which enable their coexistence in the same ecological niche. These findings demonstrate the trade-offs of ephemeral plants to maximize resource utilization and regulate their self-growth in harsh desert environments, which are the result of the gradual evolution of desert plants under long-term natural selection and interaction with the environment. In addition, the strong adaptability of *E. oxyrhinchum* make it a key player in the community structure. Finally, as ephemeral plants are important components of desert ecosystems, a variable or chronically dry climate in the future could reduce their contributions to productivity, nutrient cycling, ecosystem function, and stability.

## Figures and Tables

**Table 1:** Lifetime of four ephemeral plants under different precipitation treatments. Different lowercase letters indicate significant differences between treatments.

Species	Treatment	Lifetime (Day)
*Alyssum linifolium*	Drought	38.96 ± 0.15
	Control	39 ± 0.14
	Wet	39.24 ± 0.04
*Erodium oxyrhinchum*	Drought	50.2 ± 0.19 a
	Control	54.28 ± 0.24 b
	Wet	54.8 ± 0.41 b
*Malcolmia scorpioides*	Drought	42.68 ± 0.19 a
	Control	49.85 ± 0.32 b
	Wet	43.16 ± 0.13 a
*Hyalea pulchella*	Drought	55.52 ± 0.33
	Control	56 ± 0.30
	Wet	56.04 ± 0.31

## Data Availability

The datasets generated and/or analyzed during the current study are available from the corresponding author upon reasonable request.

## Supplementary Materials

The following supporting information can be downloaded at: https://www.mdpi.com/article/10.3390/plants12152841/s1, Figure S1: Schematic diagram of the experimental design; Table S1: Summary of a one-way ANOVA showing the effects of different precipitation treatments on four ephemeral plants. Note: * indicates *p* < 0.05; Table S2: The number of edges of the trait network for four species.
