# Supplementary material for: Responses of Ephemeral Plants to Precipitation Changes and Their Effects on Community in Central Asia Cold Desert
Source: Plants (Basel). 2023 Aug 1;12(15):2841. doi: 10.3390/plants12152841 (PMC10421208; doi:10.3390/plants12152841)

Figure S1: Schematic diagram of the experimental design.

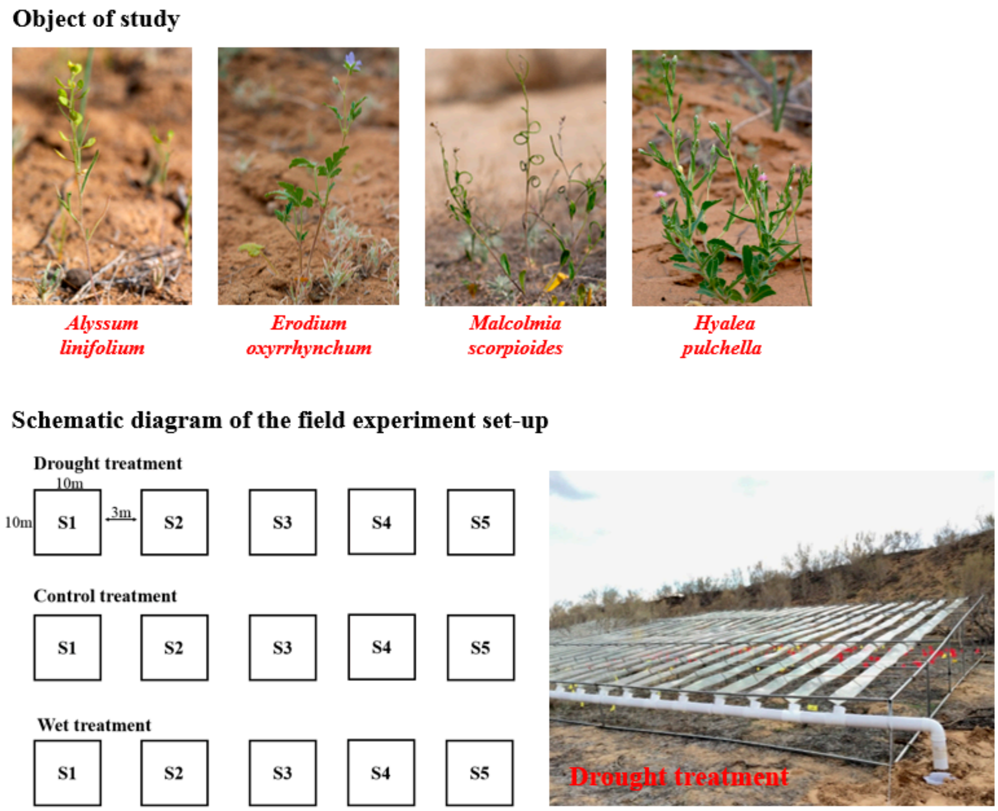

Supplement: Supplementary file 1 [file plants-12-02841-s001.zip › Figure S1.pdf]
