# Supplementary material for: Responses of Ephemeral Plants to Precipitation Changes and Their Effects on Community in Central Asia Cold Desert
Source: Plants (Basel). 2023 Aug 1;12(15):2841. doi: 10.3390/plants12152841 (PMC10421208; doi:10.3390/plants12152841)
Supplement: Supplementary file 1 [file plants-12-02841-s001.zip › Table S1.pdf]

Supplementary Table S1 Summary of a one-way ANOVA showing the effects of different precipitation treatments on four ephemeral plants. Note: * indicates $p < 0.05$.

	Alyssum linifolium		*Erodium oxyrhinchum*		*Malcolmia scorpioides*		*Hyalea pulchella*	
Plant traits	df	F	df	F	df	F	df	F
Final height	2	4.713*	2	1.97	2	10.95*	2	0.12
Lifetime	2	1.593	2	71.465	2	311.727	2	0.849
Seed production	2	0.01	2	16.16*	2	31.79*	2	18.71*
Leaf area	2	5.574*	2	29.511*	2	3.72	2	4.32*
Specific leaf area	2	11.758*	2	26.111*	2	0.14	2	7.10*
Hundred-grain weight	2	3.20	2	4.30*	2	6.00*	2	5.21*
Aboveground biomass	2	4.28*	2	114.84*	2	9.34*	2	4.83*
Importance value	2	4.20*	2	10.437*	2	0.01	2	0.31
