# Supplementary material for: Responses of Ephemeral Plants to Precipitation Changes and Their Effects on Community in Central Asia Cold Desert
Source: Plants (Basel). 2023 Aug 1;12(15):2841. doi: 10.3390/plants12152841 (PMC10421208; doi:10.3390/plants12152841)
Supplement: Supplementary file 1 [file plants-12-02841-s001.zip › Table S2.pdf]

**Supplementary Table S2** The number of edges of the trait network for four species.

| | *Alyssum linifolium* | *Erodium oxyrhinchum* | *Malcolmia scorpioides* | *Hyalea pulchella* |
|---|---|---|---|---|
| Trait | Degree | Degree | Degree | Degree |
| Survival rate | 3 | 5 | 3 | 2 |
| Height | 1 | 1 | 3 | 0 |
| Seed production | 0 | 7 | 5 | 2 |
| Leaf area | 3 | 5 | 1 | 2 |
| Specific leaf area | 2 | 4 | 0 | 0 |
| Hundred-grain weight | 4 | 2 | 2 | 4 |
| Aboveground biomass | 1 | 5 | 2 | 3 |
| Lifetime | 0 | 5 | 2 | 1 |
